# Glabralysins, Potential New β-Pore-Forming Toxin Family Members from the Schistosomiasis Vector Snail *Biomphalaria glabrata*

**DOI:** 10.3390/genes11010065

**Published:** 2020-01-07

**Authors:** Damien Lassalle, Guillaume Tetreau, Silvain Pinaud, Richard Galinier, Neil Crickmore, Benjamin Gourbal, David Duval

**Affiliations:** 1 IHPE, University of Montpellier, CNRS, Ifremer, University of Perpignan Via Domitia, 66860 Perpignan France; damien.lassalle@univ-perp.fr (D.L.); guillaume.tetreau@gmail.com (G.T.); sp23@sanger.ac.uk (S.P.); richard.galinier@univ-perp.fr (R.G.); gourbal@univ-perp.fr (B.G.); 2 School of Life Sciences, University of Sussex, Brighton BN1 9RH, UK; N.Crickmore@sussex.ac.uk

**Keywords:** pore-forming toxin, Cry toxin, invertebrate, *Biomphalaria glabrata*, host/pathogen, vector snail, innate immunity

## Abstract

*Biomphalaria glabrata* is a freshwater Planorbidae snail. In its environment, this mollusk faces numerous microorganisms or pathogens, and has developed sophisticated innate immune mechanisms to survive. The mechanisms of recognition are quite well understood in *Biomphalaria glabrata*, but immune effectors have been seldom described. In this study, we analyzed a new family of potential immune effectors and characterized five new genes that were named Glabralysins. The five Glabralysin genes showed different genomic structures and the high degree of amino acid identity between the Glabralysins, and the presence of the conserved ETX/MTX2 domain, support the hypothesis that they are pore-forming toxins. In addition, tertiary structure prediction confirms that they are structurally related to a subset of Cry toxins from *Bacillus thuringiensis*, including Cry23, Cry45, and Cry51. Finally, we investigated their gene expression profiles in snail tissues and demonstrated a mosaic transcription. We highlight the specificity in Glabralysin expression following immune stimulation with bacteria, yeast or trematode parasites. Interestingly, one Glabralysin was found to be expressed in immune-specialized hemocytes, and two others were induced following parasite exposure.

## 1. Introduction

All living organisms are confronted with environments full of complex changing populations of microorganisms and potential pathogens, engendering strong selective pressures that drive the complexification and evolution of their immune system [1,2,3]. In invertebrates, the innate immune reactions mounted to recognize and control/contain and/or eliminate microbe or parasite infections is considered to be subdivided into three steps. The first step consists of pattern recognition through microbe-associated molecular patterns (MAMPs), pathogen associated molecular patterns (PAMPs), or damage-associated molecular patterns (DAMPs) [4,5,6,7]. The second one consists of signaling pathways that regulate and control the activation of the immune response triggering the third step that consists of the production and release of effector molecules dedicated to pathogen killing [3]. These responses are mediated by the cellular response (hemocytes) and the release of humoral compounds that cooperate in the defense of the organism.

The Planorbidae snail *Biomphalaria glabrata (B. glabrata)*, constitutes an original model for studying such responses. *B. glabrata* snails face in their environment, viruses [8], bacteria [9], protozoa [10], yeast [11] nematodes [12] and various trematodes. Indeed, *Biomphalaria* sp. is mainly known for its role as vector snail of the human schistosomiasis disease for which the trematode *Schistosoma* sp. is the etiological agent, but the snail acts also as an intermediate host for numerous *Echinostoma* trematode species [13].

Therefore, it can be expected that snails have to co-evolve with these pathogen communities and should possess sophisticated immune processes to cope with them. In the past decade, the molecular basis of the *B. glabrata* innate immune response has been investigated at the genomic [14,15,16,17], transcriptomic [18,19,20], proteomic/biochemical [21,22,23,24,25,26,27] and epigenomic levels [28,29,30,31,32]. These studies have revealed that various molecules and pathways involved in immune recognition, immune regulation/activation and immune effector systems participate in a complex interplay between host and pathogen to maintain the homeostasis of the organism.

Immune recognition molecules have been investigated in *B. glabrata* snails. Among them, different families of pattern recognition receptors (PRRs) have been described, such as (i) the peptidoglycan recognition proteins (PGRPs), (ii) the toll like receptors (TLR) [33,34], (iii) calcium-dependent (C-type) lectins or C-type lectin–related proteins (CREPs) [27,35,36], and finally (iv) the fibrinogen-related proteins (FREPs) [37]. All these PRRs are essential for pathogen recognition due to their high levels of polymorphism/diversification and potential multimerization [33]. Such processes have resulted in an enlarged repertoire of putative recognition molecules that increased the potential for specific recognition, making the *B. glabrata* immune response particularly efficient at recognizing and eliminating most of the pathogens it encounters. Pathogen recognition constitutes the primary step needed to implement a coherent and efficient immune response and has been extensively investigated in *B. glabrata*, but factors involved in the immune effector pathway constitute more of a black box. After recognition of the pathogen, inducible immune effectors are activated to mount a response against the challenging pathogen. To date, the factors identified correspond to four different classes of effectors: (i) proteases or protease inhibitors, (ii) Reactive oxygen/nitrogen species (ROS/RNS), (iii) antimicrobial proteins, and (iv) cytotoxic/cytolytic toxins.

Proteases (cathepsin, elastase, and Zn metalloproteinase) and protease inhibitors (cystatin or serine protease inhibitors) can be detected after pathogen infection [38,39,40]. Such molecules might be involved in the dynamic remodeling of the extracellular matrix (ECM) and the physicochemical properties of connective tissue to impair the migration of the parasite into the snail [4]. ROS and RNS are crucial factors of the snail defense. For example, in vitro studies have revealed that H_2_O_2_ and NO contribute to the *B. glabrata* hemocyte-mediated killing of schistosome larvae. Hemocytes from *S. mansoni*-resistant snails generate significantly more H_2_O_2_ than hemocytes from susceptible snails, due to constitutive elevated levels of the mRNA encoding the copper/zinc superoxide dismutase (CuZn SOD) [15,24,41,42]. Antimicrobial peptides (AMPs) and proteins are important effectors of innate immune systems. Several studies have identified and functionally characterized antimicrobial proteins in *B. glabrata*. In particular, a LBP/BPI was identified that displayed antibacterial and anti-oomycete activities [43]. In addition, in silico studies have also identified mRNAs showing similarities with achacin from the giant African snail *Achatina fulica*, aplysianin from *Aplysia kurodai*, and a multigenic family encoding cysteine rich cationic peptides of the macin family that were named biomphamacins [44]. Changes in expression levels after immune stimulation were observed for all LBP/BPIs and three biomphamacins, further supporting their involvement in antimicrobial response [44].

Finally, concerning the cytolytic/cytotoxic toxins, their presence in *B. glabrata* has seldom been studied. The sole secreted and soluble toxin identified to date in the immune arsenal of *B. glabrata* snail is Biomphalysin [25]. This is an aerolysin-like protein belonging to β-pore-forming toxin family [25]. Biomphalysin has been shown to directly bind to *S. mansoni* sporocysts and to have a lytic activity enhanced by unidentified snail plasmatic factor(s) [25]. In addition to anti-schistosome activity, different Biomphalysin isoforms have been demonstrated to bind to gram-negative and -positive bacteria, yeast and echinostome parasites [27]. Biomphalysin forms a complex with humoral immune molecules (FREP, TEP) [45]. Altogether, this suggests that Biomphalysin is involved in pathogen clearance [27].

The β-pore-forming toxin family is an important family of proteins that can be divided into five subfamilies, the aerolysins, the haemolysins, the cholesterol dependent cytolysins (CDCs), the membrane attack complex/perforins (MACPF) and the repeated toxins (RTX). Beta-pore-forming toxins are known to form pores in targeted cell membranes to trigger cytosolic release, osmotic stress and cell lysis [46]. To facilitate the discovery of -pore-forming toxin family members, a non-targeted approach using the *B. glabrata* genome [44] was used. Five genes coding for putative toxins containing the ETX/MTX2 domain were identified and named Glabralysins. These are closely to toxins such as Cry23Aa, Cry45Aa and Cry51Aa from *Bacillus thuringiensis* and the epsilon toxins from *Clostridium perfringens* [47] which all display features of aerolysin-like -pore-forming toxins [48]. Herein, we characterize these Glabralysins as a new class of toxin proteins from the vector snail *B. glabrata*. 

## 2. Materials and Methods 

### 2.1. Ethic Statement 

Our laboratory holds permit #A66040 for experiments on animals from both the French Ministry of Agriculture and Fisheries, and the French Ministry of National Education, Research, and Technology. The housing, breeding, and care of animals utilized here followed the ethical requirements of our country. The experimenter also possesses an official certificate for animal experimentation from both French ministries (Decree #87–848, 19 October 1987). Animal experimentation followed the guidelines of the CNRS (Centre National de la Recherche Scientifique, Paris, France). The protocols used in this study have been approved by the French veterinary agency from the DRAAF Languedoc-Roussillon (Direction Régionale de l’Alimentation, de l’Agriculture et de la Forêt), Montpellier, France (Authorization #007083). 

### 2.2. Biological Material 

For in-silico approaches the *B. glabrata* BglaB1.6 genome assembly was used [49], from a strain collected in the Belo Horizonte province, district of Barreiro in the south east of Brazil. For laboratory experimental procedures, the *B. glabrata* albino strain originating from the district of Recife in the east of Brazil (named BgBRE) and maintained in the lab for several years was used. BgBRE snails 5–8 mm in diameter were collected and used for all experimental procedures. For experimental infections BgBRE snails were exposed for 1 h by whole snail soaking to various micro-organisms and parasites, the gram-negative bacterium *Escherichia coli* or the gram-positive *Micrococcus luteus*, the yeast *Saccharomyces cerevisiae* or to the trematode parasite *Schistosoma mansoni.* For SmBRE infection, adult *S. mansoni* parasites were maintained in hamsters (*Mesocricetus auratus*), parasite eggs were collected from hamster livers and were put in sterile water under light illumination for egg hatching. Then miracidia (Mi) were collected and 10 Mi per snail were used for experimental infections of BgBRE.

### 2.3. Characterization of Glabralysin Gene Organization

In order to characterize coding sequences, total RNA from a pool of 10 snails was extracted with TRIzol reagent (ThermoFisher Scientific, Paris, France) according to the manufacturer’s instructions and subsequently reverse transcribed to first strand cDNA using Maxima H Minus First Strand cDNA Synthesis Kit with dsDNase (ThermoFisher Scientific, Paris, France).

Four Glabralysin gene fragments (Gla1Aa1, Gla1Aa2, Gla1Ba1, Gla1Ca1) were obtained by running polymerase chain reaction (PCR), using GoTaq G2 Hot Start enzyme (Promega, Lyon, France). Briefly, 2.4 µL MgCl_2_ (1.5 mM), 8 µL Gotaq Buffer 5×, 0.8 µL dNTPs (10 mM), 0.2 µL Gotaq enzyme (1 unit), and 0.5 µL of primers (100 µM), for a total reaction volume of 40 µL. Primer sequences are shown in Table S1. The amplification cycling conditions were as follows: initial denaturation step at 95 °C for 6 min, followed by 40 amplification cycles composed of 30 s denaturation at 95 °C, 30 s primer annealing step at 51 °C and 1 min elongation at 72 °C. PCR reaction was ended by a final elongation step of 5 min at 72 °C. Amplicons were sequenced by Sanger method. Glabralysin gene structure (exon/intron) was determined by comparing cDNA sequences obtained from sequencing to the *B. glabrata* BglaB1.6 genome assembly using VectorBase website [49].

### 2.4. Glabralysin 3D Structure Prediction and Analysis 

Tertiary structure modeling for each Glabralysin was performed using the best aligned template against the PDB with the I-TASSER server [50,51]. The Gla1Aa1, Gla1Aa2, Gla1Ba1, Gla1Ca1 amino acid sequences were deduced from gene sequencing and whereas Gla2Aa1 was deduced from the *B. glabrata* genome annotation (release BglaB1.6). We took into account the C-score that is a confidence score for estimating the quality of predicted models by I-TASSER, and the TM-score that gives a reliable indication of the similarity with the PDB template used. This is calculated based on the significance of threading template alignments and the convergence parameters of the structure assembly simulations. A TM-score >0.5 was considered as significant to determine relevant structure prediction.

In order to visualize the variable regions in the Glabralysin structures, Consurf analysis was performed using the Consurf server alignment tool [52,53]. Deduced Glabralysin amino acid sequences were used to perform the Multiple Sequence Alignment (MSA). The Consurf analysis was carried out using default parameters. The estimated domain percentage of variability was calculated by analyzing Consurf MSA output that give a score of the most variable amino acids. The variable amino acids ratio was calculated in correlation with the domain length. The three distinct domains were delimitated in correlation with Cole and collaborator study [48].

The presence of conserved toxin-specific domains was searched in the deduced Glabralysin amino acid sequences using the Interpro server [54]. Glabralysin nomenclature was based on that developed for the *B. thuringiensis* Cry toxins [55]. This method is based on levels of protein sequence identity between candidate molecules, and the name is composed of an alpha numeric code (number, upper-case letter, lower-case letter, number). Briefly, if two sequences share more than 95% identity, they are given different last number; between 76% and 95% of identity they are given different lower-case letters, and between 45% and 76%, a different upper-case letter is given. Finally, if the % of identity is below 45% a different first number is given. According to this nomenclature, the five Glabralysins identified were named Gla1Aa1, Gla1Aa2, Gla1Ba1, Gla1Ca1, and Gla2Aa1. 

### 2.5. Phylogenetic Tree Analysis 

The protein sequences of the five Glabralysins were used as a query for a BLASTp search against the non-redundant NCBI database. The top hits belonging to different phyla were retained for further phylogenetic analysis. In addition, sequences of toxins from *Bacillus thuringiensis*, notably 2D42 used for tertiary structure prediction of Glabralysins, were included together with aerolysin-like proteins from bacteria and mollusks (including Biomphalysin from *B. glabrata*) and cytotoxin from the arthropod *Ixodes scapularis*. A total of 54 sequences were aligned using the BLOSUM62 substitution matrix [56] in ClustalW implemented in MEGA software version 7.0.26 [57] The evolutionary history was inferred by using the Maximum Likelihood method based on the JTT matrix-based model [58]. Neighbor-Join and BioNJ algorithms were applied to a matrix of pairwise distances estimated using a JTT model to perform the heuristic search from initial trees to generate the topology with the highest log likelihood value. All positions with less than 95% site coverage were eliminated from the analysis. The robustness of the tree was tested by a bootstrap analysis with 1000 replications. 

### 2.6. Quantitative Real Time PCR Assay for Glabralysins Expression Analysis 

The tissue distribution of Glabralysins, and their expression toward pathogen exposures were analyzed by preparing samples as described below. 

Six distinct biological tissues were recovered (Albumen gland (A); Stomach (S); Gut (G); Hemocytes (Hm); Foot (F) and Ovotestis (O)), a total of 8 biological replicates per tissue were recovered by dissection. All tissues were grinded individually into liquid nitrogen. Hemocytes were obtained from a pool of 20 snails, 6 replicates were used. Briefly, hemolymph was centrifuged at 5000 g for 5 min at 4 °C. Hemocyte pellets were then extracted for RNA recovery and cDNA synthesis as described above. 

For infection, snails were exposed to gram-positive, gram-negative bacteria and yeast as previously described [59]. Briefly, snails were bathed in pathogen-controlled suspension for 1 h, and then extensively washed. For SmBRE infection, each snail was individually exposed to 10 miracidia in 5 mL of pond water for 3 h. For infection procedure, 72 snails were used: 12 replicates were performed at 6 time points (3, 6, 12, 24, 48 and 96 h after infection) as previously described [60]. Uninfected (non-exposed) snails were used as control for the evaluation of the basal expression of Glabralysins.

RNA extraction was performed using TRIzol™ Reagent (ThermoFisher Scientific, Paris, France) procedure then 10 µg of RNAs were treated by DNase (TURBO DNA Free kit, (ThermoFisher Scientific, Paris, France)). Reverse transcription was done using the FIREScritp Reverse transcriptase (SolisBIODYNE, Dutscher, Brumath, France), for each reaction, 1 µg of DNase treated RNA was used. Briefly, 5 µL of RNA were incubated with 1 µL of Oligo (dT) primer (5 µM) and 10 µL of deionized water, at 65 °C for 5 min. Then, 2 µL of RT reaction buffer with DTT (1×), dNTP mix (500 µM final), 1 µL FIREScript RT (10 U/µL final), 0.5 µL of RiboGrip RNase Inhibitor (1 U/µL final) were added to the sample. Reverse transcription reactions were then performed by 30 min incubation at 50 °C in a thermocycler followed by 5 min at 85 °C to inactivate the enzyme. After cDNA synthesis, samples were 20 times diluted and stored at −80 °C until use. QPCR reactions were done using the 2× Takyon for SYBR assay from Eurogentec, Angers France. Primers used for Glabralysins’ expression are available in Table S1. Amplification efficiencies of 2 were obtained for each couple of primer used in this study.

The mean value of Ct was calculated using the second derivative maximum method of the Light Cycler 480 Software 1.5.0 (Roche). Melting curves were checked using the Tm-calling method from the same software. For Ct calculations, the S19 ribosomal protein gene was used as the housekeeping gene Glabralysin expression in tissues, and upon pathogen exposure, was normalized relative to S19.

The normality of the dataset was tested using Shapiro Wilk test, and significant differences were analyzed by pairwise Mann Whitney U test. Differences were considered significant when *p* < 0.05. 

## 3. Results

**Figure 1:**
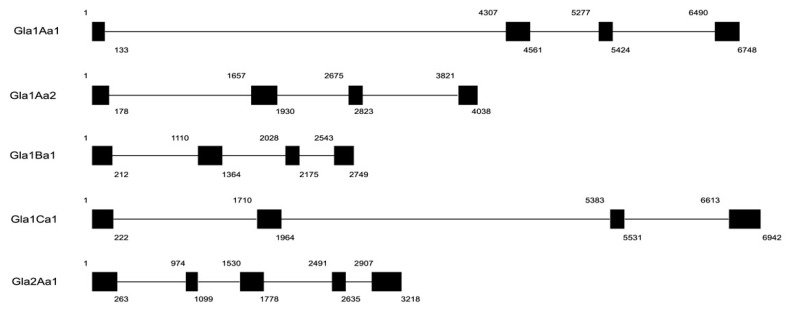
Schematic presentation of intron/exon structure of Glabralysin genes. Introns are shown as lines and exons as black boxes. Numbers in boxes represent the nucleotide position in the coding sequence.

In the present study we investigated the presence of putative new -pore-forming toxin family members from the snail *B. glabrata*. By BLASTp homology/similarity searches using the aerolysin domain of Biomphalysin (ETX/MTX2 domain) against the *B. glabrata* genome annotation (release BglaB1.6), we identified five new genes coding for toxins that have been named Glabralysins. A BLASTp search against the non-redundant NCBI database showed homology to various Cry toxins of *Bacillus thuringiensis*, this similarity prompted us to use the classification of Cry toxins from *Bacillus thuringiensis* to propose a nomenclature for the Glabralysins [55]. We showed that these five genes were positioned upon different scaffolds (Table S2). Gla2Aa1 had five exons/four introns whereas the four other Glabralysins were composed of four exons/three introns (Figure 1). Gene size and respective exon and intron positions were different for all Glabralysins (Figure 1).

**Figure 2:**
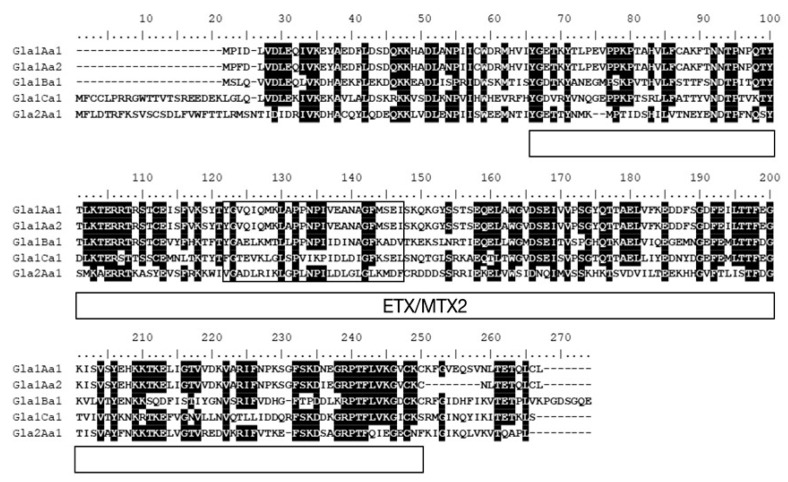
Multiple sequence alignment of Glabralysin proteins. The deduced amino acid sequences of Glabralysins were aligned using Clustal Omega program. Residues conserved in at least 80% of the aligned sequences are highlighted in black. The predicted ETX-MTX2 domain is indicated by an over lined box. The putative transmembrane domain (TMD), boxed in black, was identified using the PRED-TMBB server (prediction of transmembrane beta barrels’ proteins).

Alignment of Glabralysin primary ssequences (Figure 2) showed that identities range from 32.4% to 95.1% and similarities from 50.4% to 95.1% (Supplementary Table S3). Each Glabralysin presents a conserved ETX/MTX2 domain and a transmembrane domain that are essential for the beta barrel pore forming function (Figure 2). The ETX/MTX2 and transmembrane domain positions are relatively similar between Glabralysins (Figure 2).

**Figure 3:**
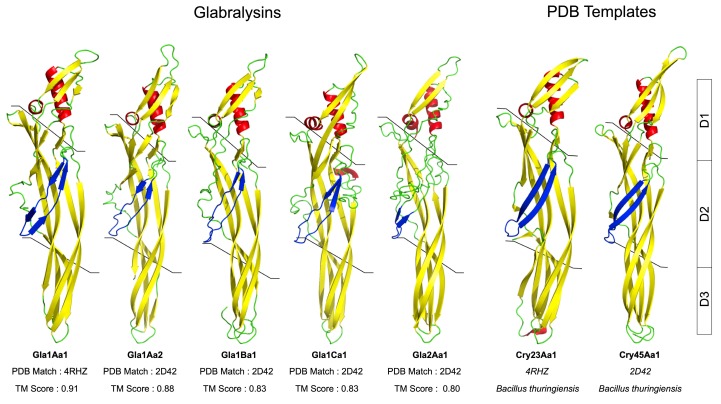
Structural prediction of the Glabralysins. Three-dimensional structure prediction of the five Glabralysins was performed using I-Tasser server with the structure of Cry23Aa1 (4RHZ) and Cry45Aa1 (2D42) as best templates. Surface representation was realized with PyMol software. Beta sheets in yellow, coils in green, alpha helix in red and TMD domain in blue. The three conserved domains (D1, D2, D3) are delimited by lines and described by boxes to the right of the figure. A TM-score was calculated between the Glabralysins and the best hit found by I-Tasser server. A score greater than 0.5 reveals significant alignment, whereas a TM-score less than 0.17 indicates random similarity.

Glabralysin 3D structure predictions were built based on Cry45Aa1 and Cry23Aa1 templates from *Bacillus thuringiensis* (2D42 for Cry45Aa1; 4RHZ for Cry23Aa1). The TM scores were higher than 0.5 and indicated significant 3D predictions. The 3D structures of the five Glabralysins revealed a high level of structural conservation between them (Figure 3). Glabralysins conserved a structural organization in three functional domains as shown for the two *Bacillus thuringiensis* toxins (Figure 3). The domain 1 (D1), is involved in receptor recognition; domain 2 (D2) takes part in the pore formation and domain 3 (D3) allows for the interaction between monomers resulting in oligomerization and pre-pore formation (Figure 3).

**Figure 4:**
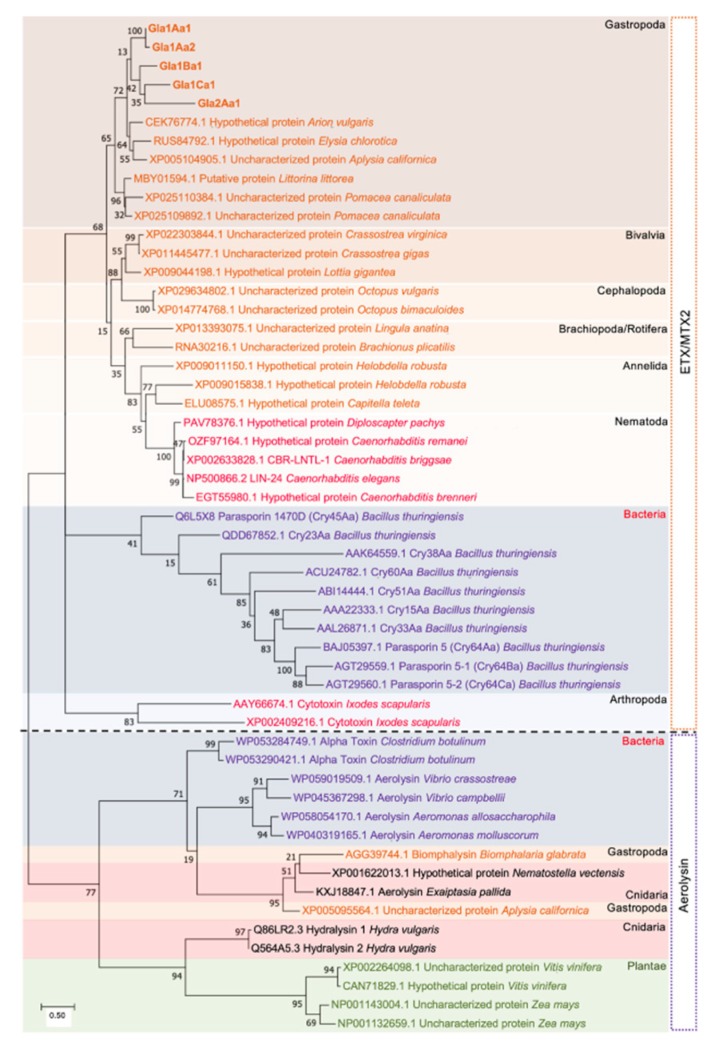
Phylogeny of the Glabralysins. Phylogenetic tree was constructed by ML method based on a JTT matrix-based model. The tree with the highest log likelihood is shown (−15,588.70). Bootstrap values higher than 0.15 are indicated at the corresponding node on the tree. The two main groups of toxins ETX and aerolysin are described by colored boxes flanked to the tree. Within the *Lophotrochozoa* group (underlined in orange), *Ecdyzozoa* (underlined in pink red), bacteria (underlined in purple), *Cnidaria* (underlined in cyan) and plantae (underlined in green). Orange degraded colored boxes represent successive clusters of *Gastropoda, Bivalvia, Cephalopoda, Brachiopoda* and *Rotifera, Annelida, Nematoda*. Purple background indicates bacteria. Red background indicates *Cnidaria* and green background regroup the *Plantae.* All *Cnidaria* species are colored in green and all *Arthropoda* in blue. For each protein, the accession number followed by the entry description and the species is indicated. Glabralysin sequences are indicated in bold. The scale bar indicates the number of substitutions per site and the tree is drawn to scale.

A phylogenetic tree was built using 54 protein sequences of ETX/MTX2 domain-containing proteins. Bacterial Cry and aerolysin toxins were used as out groups. Interestingly the Glabralysins clustered altogether, and particularly the degree of similarity between ETX/MTX2 toxins recapitulates to a significant extent the evolutionary history of species phylogenic tree (Figure 4). This is not the case for aerolysin toxins, for which phylogenetic incongruences were observed. Indeed, some aerolysins from cnidarian, bacteria, and gastropod were grouped, while other cnidarian aerolysins were closer to those from plants (Figure 4).

**Figure 5:**
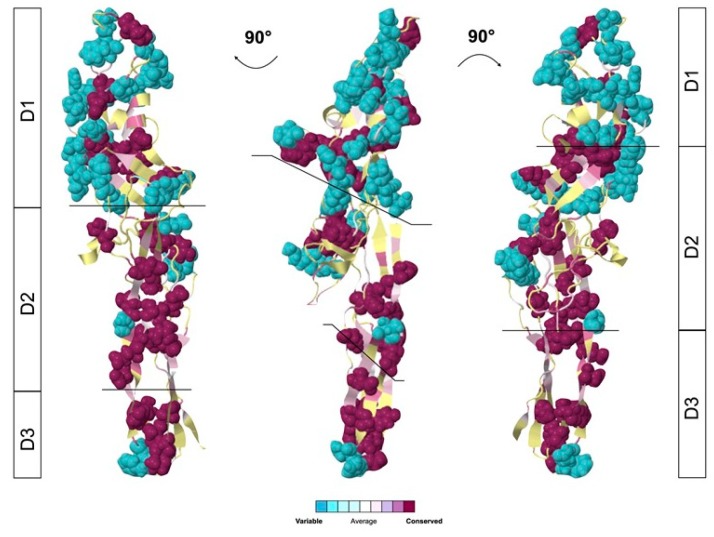
ConSurf distribution of conserved amino acids in predicted Glabralysin protein structures. Sequence conservation is plotted on the surface of the predicted structure of Gla2Aa1. Residues are colored according to the conservation, from red representing identity to cyan representing no conservation. Low reliability positions are marked yellow in the 3D structure. Different views of the same structural alignments were shown rotated by 90°.

Depending on the Glabralysins considered, a high level of variability could be observed in their primary structure alignment (Figure 2). To further characterize these variable regions, we performed a superimposition of the 3D structures of the five Glabralysins highlighting the levels of amino acid variability using CONSURF alignment tool. We identified that the most variable region is located in domain 1 (57% of variable amino-acids), while the most conserved are domains 2 and 3 (12% and 16% respectively) (Figure 5). This observation led us to suggest a potential variability in Glabralysin targets, as D1 is the recognition domain, which would be thus involved in different biological functions in *B. glabrata*.

**Figure 6:**
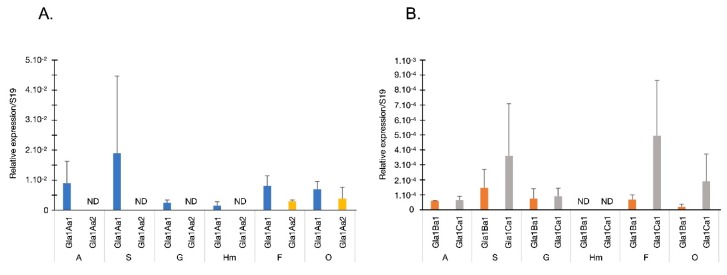
Tissue expression pattern of Glabralysins. Gene expression was quantified by RT-QPCR for Gla1Aa1 and Gla1Aa2 (**A**) and Gla1Ba1 and Gla1Ca1 (**B**). Data represent mean and standard deviation normalized expression to the S19 ribosomal protein-encoding gene. Tissues are annotated as follows: A, albumen gland; S, stomach; G, gut; Hm, hemocytes; F, head-foot; and O, ovotestis. ND: Not Detected. Expression is considered as ND when no amplification was obtained or if less than four biological replicates were detected.

**Figure 7:**
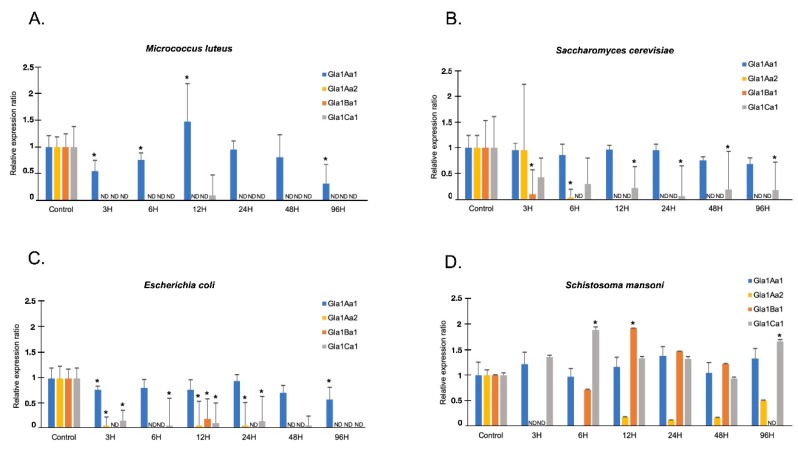
Expression of Glabralysins in response to intruders. Quantitative RT-PCR was performed from whole snails exposed to different pathogens or unexposed. Glabralysin expression was monitored at six time points (3, 6, 12, 24, 48, and 96 h) with *Micrococcus luteus* (**A**), *Saccharomyces cerevisiae* (**B**), *Escherichia coli* (**C**), and *Schistosoma mansoni* (**D**). Glabralysin (Gla1Aa1 (Blue), Gla1Ba1 (orange), Gla1Ca1 (grey), and Gla1Aa2 (yellow)) expression was normalized to S19 housekeeping gene expression and compared with the expression obtained in non-exposed snails. Non detected transcript is indicated by ND; error bars represent the SD of the relative quantification values obtained for each point. The significant difference in Glabralysins expression was evaluated according to a Mann Whitney U test. The asterisks indicate a significant difference between non-exposed and exposed snails.

To further the characterization of the Glabralysins, we performed quantitative-PCR expression assays on 6 different snail tissues (Figure 6). Moreover, *B. glabrata* snails were also exposed to gram-negative *Escherichia coli*, gram-positive *Micrococcus luteus*, the yeast *Saccharomyces cerevisae*, or the trematode *Schistosoma mansoni* (Figure 7) to quantify Glabralysin gene expression in the whole snail following immune stimulation. The first important result is that Gla2Aa1 was never detected in any tissue or infection and thus appeared never to be expressed. The analysis of tissue expression showed that Gla1Aa1 and Gla1Aa2 were expressed more than Gla1Ba1 and Gla1Ca1 (Figure 6). Concerning their tissue-specific expression, Gla1Aa1 was expressed in all the tissues tested, highest expression was observed in stomach and around 12.5 fold less in gut and hemocytes (Figure 6A). Gla1Aa2 was only expressed in foot and ovotestis (Figure 6A). Gla1Ba1 and Gla1Ca1 were expressed in all tissues except hemocytes (Figure 6B), with Gla1Ca1 being more highly expressed than Gla1Ba1 in stomach, foot and ovotestis (2.5, 7.3, and 9.8-fold respectively) (Figure 6B). It is noteworthy that Gla1Aa1 was the only Glabralysin expressed by hemocytes (Figure 6).

Concerning the expression of Glabralysins in response to an exposure to potential pathogens, Gla1Aa1 was the most expressed in all interactions (Figure 7) and appeared as never highly differentially regulated, showing a constitutive expression profile (Figure 7). Interestingly, Gla1Ba1 and Gla1Ca1 that had a similar tissue expression pattern, also displayed a very close profile of expression whatever the infection (Figure 7). Indeed, both genes were downregulated following *E. coli*, *M. luteus* and *S. cerevisiae* exposures while their expression increased by two-fold following *S. mansoni* infection at 12 h for Gla1Ba1 and at 6 h for Gla1Ca1 (Figure 7). Gla1Aa2 was down regulated, whatever the infection condition (Figure 7).

## 4. Discussion

In a previous study, we identified a cytolytic toxin called Biomphalysin with a demonstrated in-vitro anti-parasitic effect. This immune weapon was classified as a member of the—pore-forming toxin superfamily which bound to the sporocyst membrane [36] and formed pores resulting in membrane disruption and parasite death [25]. Recently, a pivotal role in snail immune memory has been proposed for this humoral effector [61]. In primed snails subjected to a secondary challenge with homologous parasites, the plasma compartment seems to support parasite clearance and as such Biomphalysin was identified as a principal circulatory component consumed in hemolymph following this immune challenge [61,62]. PFTs are most commonly described in bacteria and are known to play a role in bacterial virulence [46,63,64]. However, more and more aerolysin-like molecules are being characterized in plants and animals, venomous or not [65,66,67,68,69,70,71,72,73,74]. Interestingly, Moran and collaborators reported in metazoan organisms a high number of genes encoding aerolysin homologues acquired by horizontal gene transfer (HGT) events from bacterial sources [73,74]. Acquisition of bacterial toxin genes, often duplicated, may confer a selective advantage to the recipient organism in diverse biological functions [75] such as immunity [25,66,71,76,77], response to abiotic stress [69], predation [78] or digestion [79].

In an effort to explore new ß-PFT-like toxins in *B. glabrata*, a homology search of Biomphalysin genome sequence [44] and transcriptome [35]. For ETX/MTX2 domains was performed. We identified five new toxin genes that we named Glabralysins (Figure 1, Figure 2 and Figure 3). The Glabralysins and Biomphalysin present differences in gene structure, protein secondary and tertiary structure organization. For example, the five genes encoding Glabralysins, unlike that encoding Biomphalysin, have introns (Figure 1). Moreover, the phylogenetic analysis of their deduced amino-acid sequences showed more congruence compared to that for Biomphalysin (Figure 4) [25]. Their acquisition and domestication could therefore be older than the acquisition of Biomphalysin [80]. In the case of HGT events, a single acquisition followed by gene duplication and divergence could have occurred to acquire novel functions resulting in adaptive fitness benefits [81,82,83,84]. Secondly, conformational differences between the structures of the two types of toxins are noticeable. Biomphalysin is composed of two distinct lobes—like aerolysin related toxins, whereas Glabralysins have only a single lobe containing an ETX/MTX2 domain like toxin or some Cry toxins (Figure 3) [85]. The predicted structure of Glabralysin is dominated by strands and can be divided into three structural domains. Domain 1, the head region is composed of helices, a hairpin and a short -strand and is most likely involved in the interaction with target receptors [47,48,86,87]. Domains 2 and 3 are both sandwiches involved in membrane insertion leading to pore formation and oligomerization respectively [47,86]. Structural alignment of the Glabralysins indicates a greater dissimilarity in amino-acid composition of domain 1 suggesting potentially different capacities of recognition or binding to cellular receptors or targets (Figure 5). This domain in Bt Cry toxins shows a higher level of structural differences leading to varied levels of toxicity and/or specificity [88]. For example, Cry46 mediates its lytic activity through specific interaction with GPI-anchored proteins [89], while Cry45, unlike many β-PFTs is cholesterol independent [90]. Mutations at different tyrosine residues located in domain 1 abolish the binding ability of the epsilon toxin to MDCK cells, an epithelial cell line [91,92]. Minor differences in amino acid composition were sufficient to discriminate toxin activity as described for hydralysin 1 and 2 [78].

Based on these observations, we can hypothesize that the Glabralysin diversity identified in *B. glabrata* may result in the acquisition of new and/or diversified functions. Furthermore, their pattern of expression in snail tissues lead us to suggest that Glabralysins could displayed tissue-specific functions. Gla1Aa2 was exclusively detected in foot and ovotestis while Gla1Aa1 was the sole Glabralysin expressed in hemocytes (Figure 6). To investigate their potential involvement in the defense of the organism, we further examined the Glabralysin expression patterns in response to microbial (*E. coli*, *M. luteus* and *S. cerevisiae*) or parasite (*S. mansoni*) exposure. Gla1Aa1 was the only Glabralysin constitutively expressed whatever the pathogen used. Gla1Aa1 is thus speculated to act potentially as a sentinel effector of the innate immune system as this was demonstrated for Biomphalysin [25]. Concerning Gla1Ba1 and Gla1Ca1, their expression was slightly induced after exposition to the parasite *S. mansoni* (Figure 7). In addition, after microbial exposure (bacteria and yeast), a strong inhibition of Glabralysin gene expression (except Gla1Aa1) was observed (Figure 7). This probably reflects a balance between the different immune response pathways necessary for the activation of an efficient response directed against micro-organisms. But, an alternative hypothesis has also to be considered, the down regulation of Glabralysins could have resulted from a targeted immunosuppression derived by the micro-organisms themselves to inhibit such toxins that may have anti-microbial activities. Therefore, more efforts are now needed to decipher the precise functional role of each Glabralysin in snail physiology and defense response. First, using recombinant proteins, the hemolytic activity of Glabralysins will be investigated, and then their cytotoxic/cytolytic effects on micro-organisms or pathogens will be analyzed, as was done for Biomphalysin [25]. Moreover, as some the members of the Bt β-PFT family (the parasporins) have selective against specific human tumor cell lines [90,93,94,95,96,97], any possible anticancer activity of Glabralysins will be investigated, and any molecules showing a positive activity can be considered as potential new chemotherapeutic drugs [98,99,100].

## 5. Conclusions

In our study, we presented the first results dedicated to the description of a novel toxin family belonging to the pore-forming toxin in the schistosomiasis vector snail, *B. glabrata*. This new family called Glabralysin differ from Biomphalysin toxins in their genomic and structural organization. Each Glabralysin member has no small N-terminal lobe and therefore looks more like a classical epsilon toxin than an aerolysin-related protein. These toxins share a similar overall structure, but their domain 1 head regions—involved in receptor interaction—differ significantly in their amino-acid composition. These ETX/MTX2 toxins exhibit diverse patterns of tissue distribution, ranging from ubiquitous, such as Gla1Aa1, to tissue-specific expression. Unexpectedly, the expression level of most Glabralysin genes are strongly affected by microbial exposure and two Glabralysins are induced after *S. mansoni* infestation. Therefore, further functional characterizations are required to determine their real contribution to snail metabolism, physiology, or immune response.

## Supplementary Materials

The following are available online at https://www.mdpi.com/2073-4425/11/1/65/s1, Table S1: Primer sequences used for PCR and qPCR amplifications, Table S2: Nomenclature and genome sequence scaffold’s position for Glabralysin genes, Table S3: Sequence similarity and identity scores between Glabralysin proteins.
